# The problem of treatment gap in alcohol use disorder (AUD)

**DOI:** 10.1192/j.eurpsy.2024.37

**Published:** 2024-08-27

**Authors:** J. G. Bramness

**Affiliations:** ^1^Dep. of drug research, Oslo University Hospital, Oslo; ^2^Psychiatry, UiT The Arctic University of Norway, Tromsø, Norway

## Abstract

**Abstract:**

Alcohol use and alcohol use disorder (AUD) is related to numerous somatic and psychiatric disorders resulting in a high contribution to global burden of disease and premature death. The need to identify and treat alcohol use disorder is high. Yet there is a large treatment gap. Too few people with AUD are recognized and are being offered treatment. In some countries well under 10 percent of those with a treatable AUD are ever offered treatment. Furthermore, there is a dearth of effective treatments and relapse rates remain high. This symposium will address some topics that may change this situation.

**Disclosure of Interest:**

None Declared

